# Retraction: Diagnostic Accuracy of Point-of-Care Ultrasound in Detecting Pneumothorax: A Systematic Review and Meta-Analysis

**DOI:** 10.7759/cureus.r253

**Published:** 2026-10-09

**Authors:** Lavanya Ranganath, Deepti Gowda, Manjunath G.N.

**Affiliations:** 1 General Practice, Sri Siddhartha Medical College, Tumkur, IND; 2 Medicine, Hull University Teaching Hospitals NHS Trust, Hull, GBR; 3 Pharmacology, Sri Siddhartha Medical College, Tumkur, IND

This article has been retracted by the Editor-in-Chief. Following a reader inquiry, the pooled diagnostic accuracy estimates reported in the article were found not to be supported by the data presented, and the findings and conclusions are therefore unreliable. The authors disagree with the decision to retract.

